# Spontaneous Pneumomediastinum in Marijuana Users

**DOI:** 10.7759/cureus.45033

**Published:** 2023-09-11

**Authors:** Zeeshan Ahmad, Aveek Mukherjee, Alberto Garcia, Hamza Asif

**Affiliations:** 1 Internal Medicine, Saint Peter's University Hospital, New Brunswick, USA; 2 Internal Medicine, Rutgers Robert Wood Johnson Medical School, New Brunswick, USA; 3 Pulmonary Diseases, Saint Peter's University Hospital, New Brunswick, USA; 4 Internal Medicine, Universidad Javeriana, Bogotá, COL; 5 Pulmonology, Khyber Teaching Hospital, Peshawar, PAK

**Keywords:** marijuana and pneumothorax, atypical chest pain, macklin phenomenon, marijuana use, causes of pneumomediastinum

## Abstract

This article presents two individuals with different clinical presentations who experienced spontaneous pneumomediastinum following the chronic use of marijuana. Pneumomediastinum has been associated with marijuana use due to the prolonged inhalation and breath-holding mechanisms employed during consumption. The first case involves a 24-year-old woman with a history of anxiety and chronic marijuana use, who presented to the emergency department with atypical chest pain and shortness of breath. The second case involves a 21-year-old man with no previous medical history, who experienced acute chest pain after smoking marijuana. Both individuals exhibited signs of pneumomediastinum on imaging studies and were treated with oxygen therapy and analgesics. The cases emphasize the importance of considering pneumomediastinum in patients with atypical chest pain, particularly in chronic cannabis users.

## Introduction

Pneumomediastinum is a rare condition presented due to multiple etiologies, some of which include trauma, substance abuse, and infections. Non-traumatic pneumomediastinum is referred to as spontaneous pneumomediastinum (SPM) and has an incidence of 0.001%-0.0014% [1]. Chronic marijuana use has been linked to spontaneous pneumomediastinum (SPM) due to prolonged inhalation or breath-holding practices during use. These behaviors elevate the risk of barotrauma, resulting from excessive inflation and alveoli rupture [2]. 

The usage of marijuana and hallucinogens among young adults has had a substantial increase in 2021 when compared to the rates of five and ten years prior, this surge in usage reached levels not seen since 1988 [3]. As the prevalence of marijuana use continues to rise, it becomes crucial to recognize potential uncommon complications that may become more prevalent in the years to come. 

In this case series, we present two distinct cases involving young adults with a history of chronic marijuana use who seek medical attention in the emergency room for SPM. A 24-year-old woman with a known history of anxiety who presented an abrupt onset of chest pain and shortness of breath, and a 21-year-old male with no significant medical history who consulted for atypical chest pain, dyspnea, and palpitations. 

## Case presentation

Case 1

**Figure 1:**
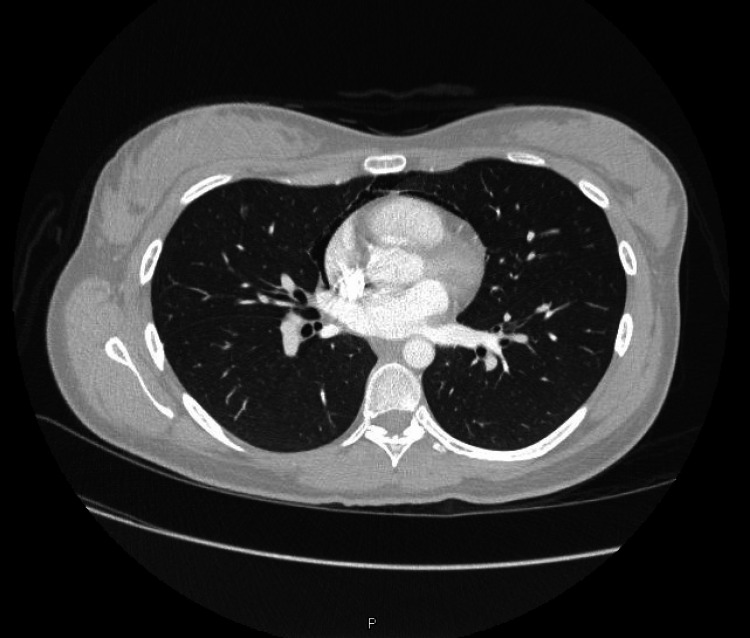
Pneumomediastinum extending from the neck to the pericardium at the base of the lungs.

A 24-year-old female with a history of anxiety and chronic marijuana use presented to the emergency department (ED) with mid-sternal chest pain described as “pressure-like” in nature for three to four hours. It was aggravated by taking deep breaths and bending forward. The pain did not radiate to any part of the body, and she also had shortness of breath, which she described as shallow. There was no inciting event preceding the chest pain except for smoking marijuana. As per past medical history, she endorsed smoking marijuana daily for five years, used lysergic acid diethylamide once in the past, and did not drink alcohol or any other substances. No significant family history was noted. Her vitals on presentation were within the normal range except for tachycardia at 104 beats per minute. Clinical examination did not reveal any subcutaneous crepitus over the chest, and the systemic exam was unrevealing. On laboratory investigations, the troponin-I levels were within normal limits (<0.03 ng/mL). A chest X-ray (CXR) was performed, and it did not reveal any abnormality. A computed tomography of the thorax with pulmonary angiogram (CTPA) showed pneumomediastinum extending from the neck to the pericardium at the base of the lungs (Figure 1). There was no hemodynamic instability, and hence the patient was closely observed. She was treated with oral analgesics and given oxygen through a non-rebreathing mask (NRM). The next day, her chest pain improved, and she was discharged home with outpatient follow-up. 

Case 2

**Figure 2:**
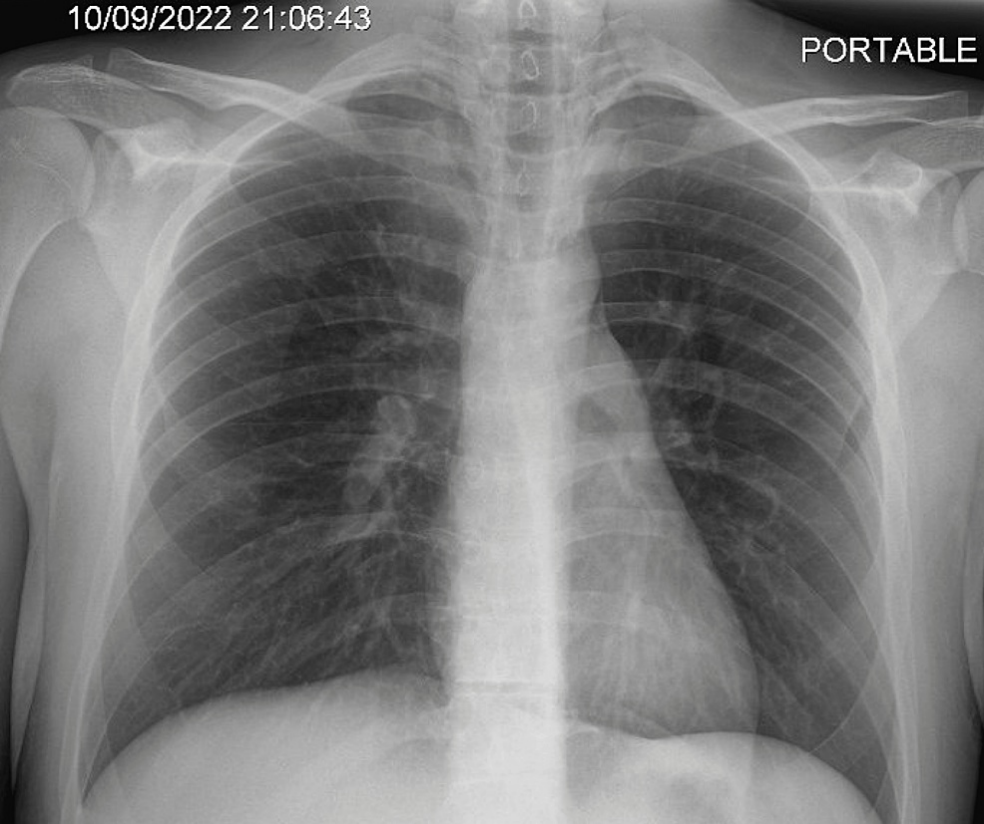
Chest X-ray reveals signs of pneumomediastinum with the presence of air surrounding the heart and in the subcutaneous tissues of the neck.

A 21-year-old man with no past medical history presented with acute-onset central, non-radiating chest pain for two hours. He had been smoking marijuana prior to the onset of the chest pain. He also complained of dyspnea and palpitations. His vital signs were notable for tachycardia at 114 beats per minute. On examination, fullness was notable in the anterior cervical area, which was non-tender. No subcutaneous crackles were auscultated, and the systemic examination was otherwise normal. Laboratory examinations were unremarkable, with normal troponin-I. The CXR revealed subtle signs of pneumomediastinum (Figure 2) with air in the neck and around the heart.

Follow-up

**Figure 3:**
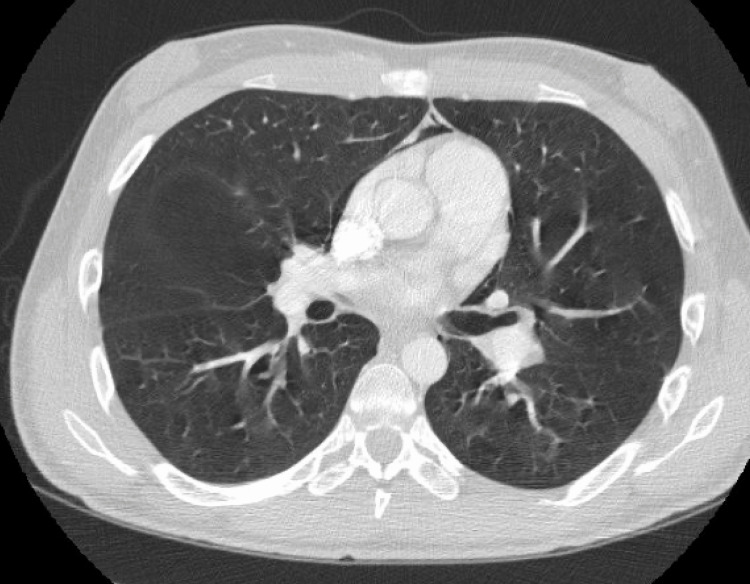
Computed tomography (CT) of the thorax pneumomediastinum with air present in the anterior and middle mediastinum.

**Figure 4:**
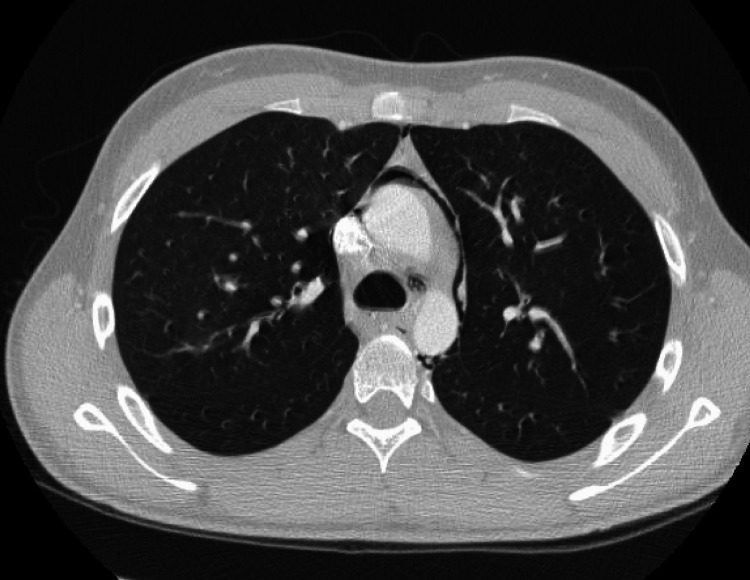
Computed tomography (CT) of the thorax pneumomediastinum extending to the cervical soft tissues.

Computed tomography (CT) of the thorax revealed pneumomediastinum with air tracking around the heart into the subcutaneous tissues of the neck (Figures 3, 4). 

The patient was started on oxygen therapy via NRM and given nonsteroidal analgesics. On clinical examination, by the next day, the normal anatomy of the anterior cervical area had been restored. A follow-up CXR revealed the resolution of the previously noted pneumomediastinum. The patient was then discharged with primary care follow-up and cautioned against further smoking of marijuana.

## Discussion

Spontaneous pneumomediastinum is a rare condition that can occur due to the inhalation route for recreational drug usage [4]. In 2019, a retrospective review of 14 cases of non-traumatic pneumomediastinum was conducted, and 66.7% were associated with marijuana use. About 50% of the cases reported daily use (case number 1), and 21% reported less than daily (case number 2). Eleven of the 14 cases presented to the emergency department with atypical chest pain and dyspnea, similar to our presenting cases. The mechanism of smoking was described in only two cases, and concurrent risk factors included vomiting (57.1%) and coughing (42.9%) [5].

The primary diagnostic modality employed in all the cases reported by Weiss et al. was initial chest X-ray imaging, followed by confirmation through chest computed tomography (CT) scans. All cases reviewed in the literature, including those presented in this report, were managed using an observational approach coupled with supportive treatments. Invasive procedures were not pursued in any of the cases [5,6]. 

The exact mechanism of pneumomediastinum secondary to marijuana use is unclear, but it is postulated that cannabis smoking may cause a sudden increase in intra-alveolar pressure leading to alveolar trauma and subsequent air into the mediastinum. This phenomenon is called the Macklin effect and can be achieved via inspiration against a closed airway (Muller’s maneuver) after forced exhalation or exhalation against a closed glottis or airway (Valsalva maneuver) [7]. Both of these maneuvers are common in Marijuana users. 

## Conclusions

Pneumomediastinum is a rare condition with a broad range of possible etiologies. In our study, both cases demonstrated atypical chest pain and dyspnea, and only marijuana consumption stands as a discernible risk factor for spontaneous pneumomediastinum. Initial X-ray imaging proved inconclusive in providing a definitive diagnosis, prompting the utilization of chest computed tomography (CT) for a final determination. Subsequent to receiving supportive care and diligent monitoring, both patients exhibited complete recovery. 

Despite the necessity for additional investigations, given the escalating incidence of marijuana usage and the concomitant consumption of other recreational substances, particularly among young adults, it becomes imperative to contemplate pneumomediastinum as a plausible diagnostic consideration for individuals presenting with atypical chest pain in the emergency department, particularly those with a history of chronic cannabis use. 

## References

[1] Loganathan S, Gungadin P, Ichim Ichim, P P (2022). EP-68 a case of spontaneous pneumomediastinum due to marijuana usage: a rare cause. Br J Surg.

[2] Paul M, Paul P, Dey D, Bhardwaj A, Paul K (2021). A case of spontaneous pneumomediastinum following ecstasy and marijuana use. Cureus.

[3] (2023). National Institutes of Health. Marijuana and hallucinogen use among young adults reached all-time high in 2021 (2022). https://www.nih.gov/news-events/news-releases/marijuana-hallucinogen-use-among-young-adults-reached-all-time-high-2021.

[4] Puri C, Rhee K, Harish VK, Slack D (2021). Marijuana induced spontaneous pneumomediastinum. J Community Hosp Intern Med Perspect.

[5] Weiss ZF, Gore S, Foderaro A (2019). Pneumomediastinum in marijuana users: a retrospective review of 14 cases. BMJ Open Respir Res.

[6] Hazouard E, Koninck JC, Attucci S, Fauchier-Rolland F, Brunereau L, Diot P (2001). Pneumorachis and pneumomediastinum caused by repeated Müller's maneuvers: complications of marijuana smoking. Ann Emerg Med.

[7] Macklin CC (1939). Transport of air along sheaths of pulmonic blood vessels from alveoli to mediastinum: clinical implications. Arch Intern Med.

